# Spatial and temporal variability of radon and meteorological parameters in the dwellings of Villy

**DOI:** 10.1038/s41598-026-58476-0

**Published:** 2026-06-18

**Authors:** Cedric E. Beogo, Wepari C. Yaguibou, Luc T. Bambara

**Affiliations:** 1 Thomas SANKARA University, 12 BP 417 Ouagadougou 12, Ouagadougou, Burkina Faso; 2 Laboratoire de Materiaux et Environnement (LAME), Joseph KI-ZERBO University, Ouagadougou, Kadiogo, 03 BP 7021 Burkina Faso; 3 Institut des Sciences et de Technologie, Ecole Normale Supérieure, Ouagadougou, Burkina Faso

**Keywords:** Radon, Indoor air, Atmospheric parameters, Dwellings, Environmental sciences, Physics

## Abstract

Radon is the leading cause of lung cancer after smoking, and its concentration can reach critical levels when it accumulates inside a dwelling through pores and cracks connecting the basement to the living space. This concentration can be influenced by other factors, such as atmospheric parameters. The values of these variables were measured inside 21 dwellings in the village of Villy. These variables are measured using a corentium pro equipped with a silicon detector to quantify radon and four sensors to measure meteorological parameters and detect movement during data acquisition. Inside each dwelling, a short-term measurement is taken for 48 h. The results show that radon-222 concentrations ranged from$\:\:13.4\pm\:5.4$ to $\:122.4\pm\:28.5\:Bq\cdot\:{m}^{-3}$, with an average value of $\:45.15\pm\:12.80\:Bq\cdot\:{m}^{-3}$. The inside temperature ranged from 32.3$\:\pm\:$0.5 to 38.8$\:\pm\:0.5$ °C, with an average value of 36$\:\pm\:0.5$ °C. Humidity ranged from 12.5$\:\pm\:1.1$ to 40.4$\:\pm\:2.3$ %rh, with an average value of 28.1$\:\pm\:1.8$ %rh and pressure ranged from 97.3175$\:\pm\:1$ to 97.7171$\:\pm\:1$ kPa, with an average of 97.5350$\:\pm\:1$ kPa. During the day, radon concentration is highest at 4 a.m. and lowest at 11 a.m. This concentration follows a two-way pattern. It increases between midnight and 7 a.m. and between 6 p.m. and midnight, then decreases from 7 a.m. to 6 p.m. The spatiotemporal distribution reveals that atmospheric parameters influence radon concentration. A decrease in temperature corresponds to an increase in humidity and pressure, leading to an increase in indoor radon concentration. Conversely, an increase in temperature corresponds to a decrease in humidity and pressure, leading to a decrease in indoor radon concentration.

## Introduction

Radioactivity exists everywhere in the environment. It is characterized by the emission of ionizing radiation by a radionuclide. This ionizing radiation interacts with matter, potentially altering DNA molecules in living organisms. There are three naturally occurring radioactive decay series defined by ^238^U, ^235^U and ^232^Th. There half-lives can reach several billion years which explains their abundance in the environment. Each naturally occurring radioactive series contains an isotope of radon. Rn-222 is from ^238^U series with a half-life of 3.82 days, thoron or ^220^Rn is from ^232^Th series with a half-life of 55.6 s and actinon or ^219^Rn is an isotope of radon produced in the ^235^U decay chain. It has a short half-life together with thoron, in the order of 3.9 s. Because of their short half-life, thoron and actinon concentration are very often neglected. Inside a dwelling, ^222^Rn has a half-life long enough to accumulate and reach concentrations that could be dangerous to health. This concentration is influenced by the building materials, which may contain uranium; the ventilation system of the building; the geological nature of the soil, which may contain relatively high levels of radioactivity like in Serbia where ^226^Ra concentration reached$\:\:295\:Bq\cdot\:{kg}^{-1}$^1^; and the condition of the excavation, which may or may not allow gases from the subsoil to pass into the building through cracks or pores. Atmospheric parameters such as temperature and humidity also influence radon concentration, but the relationship between these factors is not always clear and depends on the study area. When radon is inhaled, a large portion is exhaled and expelled from the body, while a small fraction remains trapped inside the lungs and respiratory tract, causing irradiation through direct decay or the decay of its decay products. This irradiation can lead to several diseases, including lung cancer. The World Health Organization (WHO) has recognized radon as the leading cause of lung cancer after smoking and the primary source of human exposure to radioactivity^2,3^. The radiological risks associated with exposure to radon depend on its high concentration in confined and frequently used areas such as dwellings, schools and offices. An assessment of radon concentration levels in these environments is necessary.

In Burkina Faso, studies have shown that there are some places in some localities where radon concentration is higher than the reference value established by the WHO even if the average values remain always within the acceptable limit. Radon concentration have been measured at Koudougou inside dwellings, at Ouagadougou inside residential buildings, students’ residencies and some offices of the “Ecole Normal Superieur” and in some schools of Kaya^4–8^. Since radon is a decay product of the three radioactive decay series, their high concentrations in an area could influence radon levels. In the north-eastern part of Burkina Faso, studies have shown relatively high levels of natural radioactivity. In the study conducted by^9^, radiological anomalies were identified in the localities of Pougbone, Nyapsi, and Gourgnel for ^232^Th, ^238^U and ^40^K, respectively. For the Nyapsi uranium anomaly, measurements revealed a ^238^U concentration three times higher than the world average^10^. In the west-central region, a study of gamma dose rate distribution, particularly in the rural communities of Kindi, Ramongo, and Villy, revealed the existence of radiological anomalies in this part of the country^11^. In this previous study, the average dose rate recorded in the locality of Villy was significantly higher than in other localities.

Natural radioactivity is relatively high in the village of Villy, and it constitutes the primary source of radon that can accumulate inside homes and reach critical concentration levels. However, no study has yet determined the radon concentration in radiological anomalies in Burkina Faso. Such a study is necessary to assess the impact of radioactivity on public health. The radon concentration inside homes in Villy is therefore a major concern in this study. Several studies have been conducted in this area in other parts of the country and elsewhere in the world^12–18^. However, research examining the temporal evolution of radon and its influence on meteorological parameters is somewhat scarce. This prove the importance of determining periods of day when radon concentrations are at their highest, as well as the meteorological parameters that influence its behaviour.

The present study aims to analyse the influence of meteorological parameters on radon concentration inside dwellings over time and space. This involves measuring radon concentration, temperature, humidity, and pressure inside dwellings in the study area and comparing their spatial and temporal variability.

## Materials and methods

### Study area

The study area is located in the west-central part of Burkina Faso. It is a predominantly flat region with a crystalline bedrock. Soils are mostly ferruginous and sandy. Buildings are mostly constructed with local materials and feature a rural architectural style. The village of Villy is located in the Boulkiemde province, a few kilometres from the city of Koudougou, the regional capital of the West-central part of Burkina Faso. Located at approximately 89 km from Ouagadougou, the capital of Burkina Faso, and about 21 km from Koudougou, Villy is bordered to the north by Pouedogo, to the south by Rana, to the east by the INERA research station, and to the west by Kassou. The climate is Sudano-Sahelian, characterized by a dry and cool season from November to February, a hot season from March to May, and a rainy season from June to October. Minimum temperatures are around 25 °C, while maximum temperatures can reach 45 °C. Rainfall in the west-central region is moderate, averaging around 841 mm/year, while the south of the country can reach an average of 1100 mm/year.

**Fig. 1:**
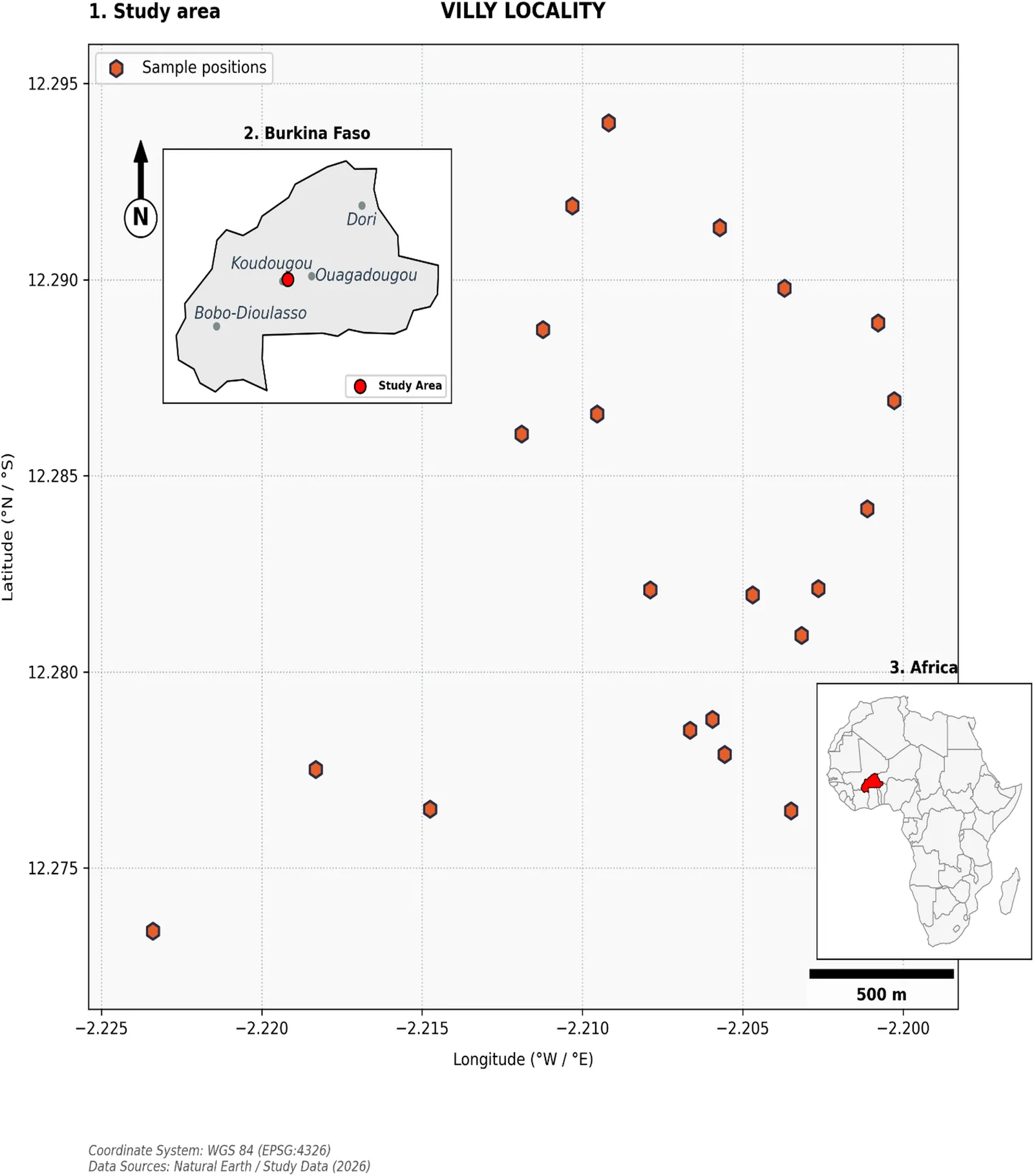
Study area showing the locations of dwellings. The map was generated by the authors using Python (version 3.14.5, python.org) with the Geopandas library (version 1.1.3, geopandas.org).

Figure 1 illustrates the study area, showing the locations of the various dwellings. The study area is specified on the map of Burkina Faso, whose position is located on the map of Africa. Dwellings were chosen at sufficiently regular intervals to ensure statistical representativeness of the data. The observed gaps between selected dwellings represent uninhabited locations. The village of Villy is subdivided into eight (8) parts, which are: Villy-Centre, Villy-Ronsin, Villy-Godin, Villy-Nadioulou, Villy-Rana, Villy-Siguivoussé, Villy-Yalgatenga and Villy-Ralmou. The present study area is specifically concerned by the Villy-Centre entity. Raw earth dwellings are approximately 2.6 m high while cement dwellings are between 2.8 and 3.0 m. Most of the houses have steel roofs and windows and consist of a single room. Some houses, mainly those built with raw earth, have thatched roofs. For houses with several rooms, the equipment is placed on the floor, and when it is a single room, the equipment is placed directly in the bedroom. This is not very convenient because the corentium pro device emits light signals that can disturb the sleep of the occupants. This constitutes a limiting factor, making it difficult to acquire data over a long period with this type of equipment.

### Measurement of radon concentration in dwellings

#### Description of the measuring device

The device used to measure radon concentration is a calibrated corentium pro from Airthings which allows the acquisition of radon concentration, temperature, pressure and humidity. It is a lightweight, portable, battery-operated continuous radon monitor (CRM) certified by the Canadian National Radon Proficiency Program (C-NRPP). It features four silicon photodiodes located inside four radon chambers. These passive diffusion chambers are where radon is trapped, identified and quantified by the alpha spectrometry method. In addition, the corentium pro is equipped with temperature, humidity, pressure, and tilt sensors to detect whether the device has been moved during the acquisition period. The results obtained from each acquisition can be stored in the device’s memory for five years, and each recorded result corresponds to one hour of data acquisition for radon concentration, humidity, temperature, and pressure. The measurement uncertainty for radon concentration is $\:7\%\:\pm\:5\:Bq\cdot\:{m}^{-3}$ after 24 h of data acquisition and $\:5\%\:\pm\:2\:Bq\cdot\:{m}^{-3}$ after one week of data acquisition. The uncertainty for temperature, humidity and pressure are$\:\:\pm\:0.5\:^\circ\:C$, $\:0.5\%rh\pm\:4.5\%$ and $\:\pm\:1\:kPa\:$ respectively. To ensure accurate results, a minimum data acquisition period of 48 h is required. The measurement ranges for radon, temperature, humidity and pressure are * 0* to $\:{10}^{5}\:Bq\cdot\:{m}^{-3}$, 4 to 40 °C, 5 to $\:85\:\%rh$ and 50 to 110 * kPa*, respectively.

The minimum detectable activity can be calculated by applying the Curie method given by the following formula:

$$\:MDA=\frac{2.71+3.29\sqrt{B}}{S\cdot\:t}$$

where $\:B\:$ is the background in number of counts, * t* is the measurement duration in hour, * S* is the detector sensibility.

The corentium pro calibration factor is about 0.954 and the background exposure for 24 h is $\:8\:Bq\cdot\:{m}^{-3}$. The detector sensibility is equal to:

$$\:S=0.1\times\:0.954=0.0954$$

The background in number of counts is then equal to:

$$\:B=8\times\:0.0954\times\:24\approx\:18.32\:$$

The Minimum Detectable Activity value in $\:Bq\cdot\:{m}^{-3}$ is given by:

$$\:MDA=\frac{2.71+329\times\:\sqrt{18.32}}{0.0954\times\:24}\approx\:7.33\:$$

The expanded uncertainty is given by the following expression:

$$\:U=k\times\:\frac{\sigma\:}{\sqrt{n}}$$

Where k = 2 is the coverage factor for a 95% confidence level, $\:\sigma\:$ is the standard deviation of measured samples and * n=42* is the total number of sample. In the present study there are 21 dwellings and two days measurement for each dwelling gives a total of 42 measures for each hour.

#### Indoor radon-222 measurements

The measurement procedure began by identifying dwellings using a grid across the study area, with approximately 1 km intervals between consecutive grid nodes. Next, the dwelling closest to each of the 21 resulting nodes was selected. Some dwellings (28%) built with raw earth, have thatched roofs, and no terraces, while the remaining 72% are built with cement and have terraces. It is noted that none of these dwellings have a ventilation system. The only way to renew the air inside dwellings is by opening doors and windows. To perform a measurement, the monitor is placed approximately 1 m above the floor inside the dwelling, and data acquisition is programmed for 48 h. At the end of this period, results are recorded and sent to the server for further analysis. Measurements have been performed from March 15th to April 1st, 2025.

Most dwellings in the study area consist of a single room containing the measuring device. With the resident’s agreement, a location is chosen within the house about one meter from the wall, and the equipment is placed on a table approximately one meter high. The number of inhabitants per house varies considerably, and during the hot season, the interior of the house is less occupied. Mostly occupants are inside dwellings from 6 p.m. to 5 a.m.

## Statistical analysis of data

The Shapiro-Wilk normality test was applied to the data to verify whether radon concentrations and atmospheric parameters follow a Gaussian distribution. To examine this normality, a qq-plot was performed on each variable. The arithmetic mean, median, and standard deviation were determined. To examine the behaviour of the data relative to the Gaussian distribution, a normality test could be applied to the logarithmic transform of the data if necessary.

To study the trends in radon concentrations with atmospheric parameters inside homes, a Spearman correlation coefficients were determined and represented on a heat map for the different variables.

### Results and discussion

#### Indoor ^222^Rn activity concentration in dwellings

**Fig. 2:**
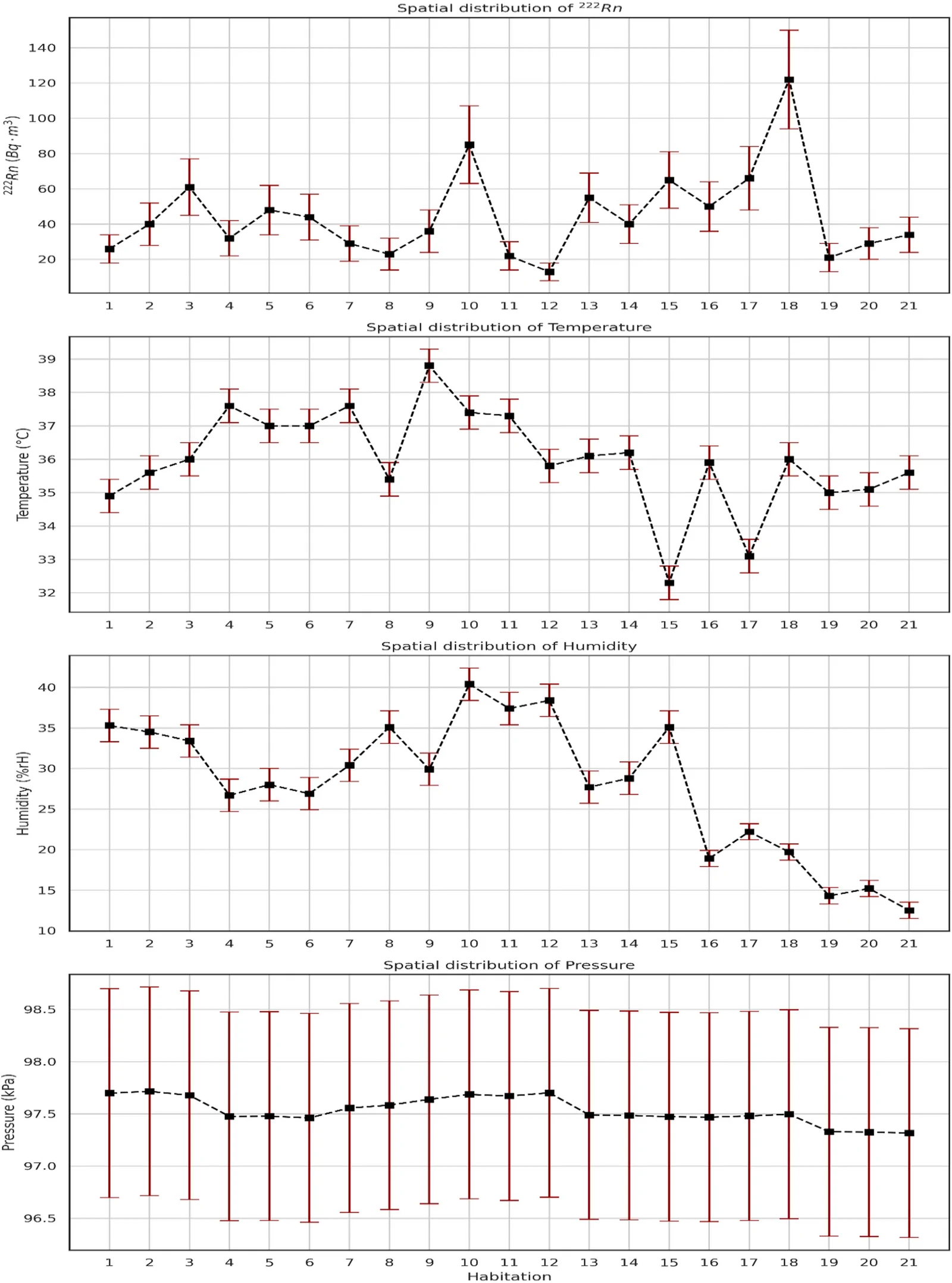
Spatial distribution of radon activity concentration, temperature, humidity and pressure expressed with expanded uncertainty (k = 2 and 95% confidence interval) in 21 dwellings in the village of Villy.

Radon concentration, temperature, humidity and pressure were measured in 21 dwellings in the village of Villy and results are summarized in Table 1. Radon activity concentration ranged from $\:13.4\pm\:5.4$ to $\:122.4\pm\:28.5\:Bq\cdot\:{m}^{-3}$, with an average value of $\:45.15\pm\:12.8\:Bq\cdot\:{m}^{-3}$. The standard deviation is about $\:25.19\:Bq\cdot\:{m}^{-3}$ which denotes a significant variability of radon spatial distribution. Indoor temperature ranged from 32.3$\:\pm\:0.5$ to 38.8$\:\pm\:0.5$ °C, with an average value of 35.98$\:\pm\:0.50$ °C. Humidity ranged from 12.5$\:\pm\:1.1$ to 40.4$\:\pm\:2.3$ %rh, with an average value of 28.13$\:\pm\:1.77$ %rh, and pressure ranged from 97.3175$\:\pm\:1$ to 97.7171$\:\pm\:1$ kPa, with an average value of 97.5350$\:\pm\:1$ kPa. Figure 2 shows the spatial distribution of radon activity concentration, temperature, humidity, and pressure values. It is noted that radon varies considerably from one dwelling to another. Only one dwelling has a radon concentration exceeding 100 $\:Bq\cdot\:{m}^{-3}$, six dwellings have radon concentrations between 50 and 100 $\:Bq\cdot\:{m}^{-3}$ and the remaining 14 dwellings have radon concentrations below 50 $\:Bq\cdot\:{m}^{-3}$. Dwellings 19, 20, and 21, located next to each other, have the lowest pressure and humidity levels, which leads to low radon concentrations. These dwellings are built of cement and each has a terrace that acts as a barrier to radon diffusion from the subsoil into the dwelling. Conversely, house 18 has the highest radon concentration with relatively low humidity and pressure values. Furthermore, the temperatures inside the four houses (18, 19, 20, and 21) are of the same order of magnitude. The higher radon concentration could therefore be explained by the geological nature of the soil, since a heterogeneous distribution of natural radionuclides can occur within the same locality. There are likely faults in the underlying geology in certain areas that facilitate radon diffusion and its accumulation inside some dwellings.

**Table 1:** Heterogeneous distribution of radon concentration expressed with expanded uncertainty (k = 2 and 95% confidence interval) inside 21 dwellings in the village of Villy and its evolution with meteorological parameters (March 2025).

House	Radon ($\:\boldsymbol{B}\boldsymbol{q}\cdot\:{\boldsymbol{m}}^{-3}$)	Temperature (°C)	Humidity (%rh)	Pressure (kPa)
COVI001	$\:26.5\pm\:7.9$	34.9$\:\pm\:0.5$	35.3$\:\pm\:2.09$	97.6995$\:\pm\:1$
COVI002	$\:40.5\pm\:11.9$	35.6$\:\pm\:0.5$	34.5$\:\pm\:2.05$	97.7171$\:\pm\:1$
COVI003	$\:60.8\pm\:15.9$	36$\:\pm\:0.5$	33.4$\:\pm\:2$	97.6791$\:\pm\:1$
COVI004	$\:31.6\pm\:10.0$	37.6$\:\pm\:0.5$	26.7$\:\pm\:1.7$	97.4771$\:\pm\:1$
COVI005	$\:48.5\pm\:14.1$	37$\:\pm\:0.5$	28$\:\pm\:2$	97.4792$\:\pm\:1$
COVI006	$\:44.4\pm\:12.8$	37$\:\pm\:0.5$	26.9$\:\pm\:1.7$	97.4627$\:\pm\:1$
COVI007	$\:29.4\pm\:10.2$	37.6$\:\pm\:0.5$	30.4$\:\pm\:1.9$	97.5575$\:\pm\:1$
COVI008	$\:23.5\pm\:9.3$	35.4$\:\pm\:0.5$	35.1$\:\pm\:2.1$	97.5841$\:\pm\:1$
COVI009	$\:36.5\pm\:12.3$	38.8$\:\pm\:0.5$	29.9$\:\pm\:1.8$	97.6407$\:\pm\:1$
COVI010	$\:85.3\pm\:22.1$	37.4$\:\pm\:0.5$	40.4$\:\pm\:2.3$	97.6875$\:\pm\:1$
COVI011	$\:21.6\pm\:8.1$	37.3$\:\pm\:0.5$	37.4$\:\pm\:2.2$	97.6726$\:\pm\:1$
COVI012	$\:13.4\pm\:5.4$	35.8$\:\pm\:0.5$	38.4$\:\pm\:2.2$	97.7032$\:\pm\:1$
COVI013	$\:55.5\pm\:14.1$	36.1$\:\pm\:0.5$	27.7$\:\pm\:1.7$	97.4912$\:\pm\:1$
COVI014	$\:40.5\pm\:10.6$	36.2$\:\pm\:0.5$	28.8$\:\pm\:1.8$	97.4866$\:\pm\:1$
COVI015	$\:65.5\pm\:16.4$	32.3$\:\pm\:0.5$	35.1$\:\pm\:2.1$	97.474$\:\pm\:1$
COVI016	$\:50.5\pm\:13.8$	35.9$\:\pm\:0.5$	18.9$\:\pm\:1.3$	97.4699$\:\pm\:1$
COVI017	$\:66.4\pm\:17.8$	33.1$\:\pm\:0.5$	22.2$\:\pm\:1.5$	97.4811$\:\pm\:1$
COVI018	$\:122.4\pm\:28.5$	36$\:\pm\:0.5$	19.7$\:\pm\:1.4$	97.4977$\:\pm\:1$
COVI019	$\:21.4\pm\:8.2$	35$\:\pm\:0.5$	14.3$\:\pm\:1.1$	97.3303$\:\pm\:1$
COVI020	$\:29.5\pm\:9.2$	35.1$\:\pm\:0.5$	15.2$\:\pm\:1.2$	97.3264$\:\pm\:1$
COVI021	$\:34.4\pm\:10.1$	35.6$\:\pm\:0.5$	12.5$\:\pm\:1.1$	97.3175$\:\pm\:1$
Min	$\:13.4\pm\:5.4$	32.3$\:\pm\:0.5$	12.5$\:\pm\:1.1$	97.3175$\:\pm\:1$
Max	$\:122.4\pm\:28.5$	38.8$\:\pm\:0.5$	40.4$\:\pm\:2.3$	97.7171$\:\pm\:1$
Average	$\:45.15\pm\:12.8$	**35.98** $\:\pm\:0.5$	**28.1** $\:\pm\:1.8$	**97.5350** $\:\pm\:1$
Std	* 25.19*	1.49	8.26	0.13

Bold values in Table 1 represent the average values of radon concentration, temperature, humidity and pressuremeasured in the dwellings of the study area.

Most of the dwellings (72%) in the study area are built of cement and have a terrace. This could explain the relatively low radon concentrations, as the terrace can act as a physical barrier between the subsoil and inside the dwellings. It should be noted that the house with the highest radon concentration, even though it is built of cement, does not have a terrace or a mechanical ventilation system. The only way to renew the indoor air is through natural ventilation caused by opening doors and windows. This can contribute to radon accumulation if the house is not regularly ventilated through these openings.

Table 2 illustrates a comparison of some results obtained in other countries with those of this study. It is noted that radon concentration in the present study is relatively low. It is similar to the results obtained for basements of dwellings in Kampala, Uganda and slightly higher than the ground, first and second floor of the same locality^19^. Radon concentration in the present study is also slightly higher than those obtained in the localities of Karbala in Iraq, Tunisia, Egypt and Kaya in Burkina Faso^20–23^. However, it is significantly lower than the average concentration measured in dwellings in Bulgaria, the Greater Accra region and the village of Buddonithanda in India^24–26^.

**Table 2:** Comparison of average radon concentrations measured inside homes in the present study with various countries around the world.

City/Country	Building		Exposure time (days)	Radon ($\:\boldsymbol{B}\boldsymbol{q}\cdot\:{\boldsymbol{m}}^{-3}$)		Reference
	Number	Type		Range	Average	
Burkina Faso (Villy)	21	dwelling	2	13–122	45	Current study
Uganda (Kampala)	10	dwelling	1	15.3–188.2	71.7	^19^
Iraq (Karbala)	20	dwelling	90	21.79–43.38	32.34	^20^
Bulgaria	2778	dwelling	365	11–1314	111	
Tunisia	69	dwelling	60	BDL − 285	39	^21^
Egypt	45	dwelling	365	3–97	30.9	^22^
Ghana (Accra)	95	dwelling	90	36–92	51	^25^
India (Buddonithanda)	22	dwelling	90	14–675	122	^26^
Burkina Faso (Kaya)	21	dwelling	1	3.78–198.22	25.68	^23^

### Indoor ^222^Rn activity concentration temporal variability

Table 3 presents the diurnal variation of radon, temperature, humidity and pressure inside 21 dwellings of Villy. The average radon concentration ranged from $\:29.71\pm\:6.47Bq\cdot\:{m}^{-3}$ recorded at 11:00 AM to $\:72.76\pm\:17.18\:Bq\cdot\:{m}^{-3}$ recorded at 4:00 AM. The minimum values of atmospheric parameters are 97.33$\:\pm\:0.04$ kPa recorded at 5:00 PM, 31.73$\:\pm\:0.55$ °C recorded at 7:00 AM, and 23.26$\:\pm\:2.42$ %rh recorded at 6:00 PM for pressure, temperature, and humidity respectively. The maximum values for pressure, temperature and humidity were 97.75$\:\pm\:0.04$ kPa recorded at 10:00 AM, 40.10$\:\pm\:0.73$ °C recorded at 4:00 PM and 33.79$\:\pm\:3.55\:$%rh recorded at 9:00 AM, respectively.

**Table 3:** Diurnal variability of radon concentration inside dwellings in the village of Villy with meteorological parameters expressed with expanded uncertainty (k = 2 and 95% confidence interval) from mean values of 21 dwellings.

Time (h)	Radon ($\:\boldsymbol{B}\boldsymbol{q}\cdot\:{\boldsymbol{m}}^{-3}$)	Pressure (kPa)	Temperature (°C)	Humidity (%rh)
1	$\:63.18\pm\:14.35$	97.56$\:\pm\:$0.04	34.64$\:\pm\:$0.53	28.44$\:\pm\:2.59$
2	$\:68.64\pm\:15.84$	97.51$\:\pm\:$0.04	34.13$\:\pm\:$0.53	28.98$\:\pm\:$2.72
3	$\:59.88\pm\:14.42$	97.47$\:\pm\:$0.04	33.55$\:\pm\:$0.54	29.54$\:\pm\:$2.86
4	$\:72.76\pm\:17.18$	97.46$\:\pm\:$0.04	32.97$\:\pm\:$0.56	30.21$\:\pm\:$3.00
5	$\:62.07\pm\:14.89$	97.49$\:\pm\:$0.04	32.47$\:\pm\:$0.58	30.88$\:\pm\:$3.15
6	$\:65.56\pm\:18.45$	97.53$\:\pm\:$0.04	31.96$\:\pm\:$0.60	31.89$\:\pm\:$3.33
7	$\:60.60\pm\:13.76$	97.60$\:\pm\:$0.04	31.73$\:\pm\:$0.55	33.04$\:\pm\:$3.51
8	$\:46.70\pm\:10.57$	97.68$\:\pm\:$0.04	32.27$\:\pm\:$0.48	33.60$\:\pm\:$3.63
9	$\:36.82\pm\:8.24$	97.74$\:\pm\:$0.04	33.25$\:\pm\:$0.46	33.79$\:\pm\:$3.55
10	$\:37.61\pm\:7.86$	97.75$\:\pm\:$0.04	34.48$\:\pm\:$0.50	32.98$\:\pm\:$3.22
11	$\:29.71\pm\:6.47$	97.74$\:\pm\:$0.04	35.84$\:\pm\:$0.57	31.49$\:\pm\:$3.00
12	$\:33.03\pm\:8.54$	97.67$\:\pm\:$0.04	37.16$\:\pm\:0.68$	29.77$\:\pm\:$2.77
13	$\:37.85\pm\:11.46$	97.58$\:\pm\:$0.05	38.28$\:\pm\:$0.77	28.45$\:\pm\:$2.72
14	$\:41.73\pm\:12.63$	97.47$\:\pm\:$0.05	39.24$\:\pm\:$0.81	26.61$\:\pm\:$2.64
15	$\:47.73\pm\:20.44$	97.39$\:\pm\:$0.05	39.89$\:\pm\:$0.79	25.25$\:\pm\:$2.49
16	$\:45.08\pm\:12.18$	97.34$\:\pm\:$0.05	40.10$\:\pm\:$0.73	24.36$\:\pm\:$2.41
17	$\:38.41\pm\:11.85$	97.33$\:\pm\:$0.04	39.96$\:\pm\:$0.69	23.81$\:\pm\:$2.44
18	$\:31.18\pm\:7.76$	97.36$\:\pm\:$0.04	39.61$\:\pm\:$64	23.26$\:\pm\:$2.42
19	$\:36.11\pm\:8.46$	97.41$\:\pm\:$0.04	38.88$\:\pm\:$0.61	23.54$\:\pm\:$2.23
20	$\:41.44\pm\:10.34$	97.46$\:\pm\:$0.04	38.05$\:\pm\:$0.59	24.19$\:\pm\:$2.18
21	$\:44.38\pm\:10.44$	97.53$\:\pm\:$0.04	37.30$\:\pm\:$0.54	24.96$\:\pm\:$2.23
22	$\:48.14\pm\:13.27$	97.59$\:\pm\:$0.04	36.60$\:\pm\:$0.52	25.85$\:\pm\:$2.26
23	$\:61.03\pm\:13.7$	97.61$\:\pm\:$0.04	35.94$\:\pm\:$0.51	26.80$\:\pm\:$2.40
24	$\:66.26\pm\:14.56$	97.60$\:\pm\:$0.04	35.29$\:\pm\:$0.53	27.63$\:\pm\:$2.48
Min	$\:29.71\pm\:6.47$	97.33$\:\pm\:$0.04	31.73$\:\pm\:$0.55	23.26$\:\pm\:$2.42
Max	$\:72.76\pm\:17.18$	97.75$\:\pm\:$0.04	40.10$\:\pm\:$0.73	33.79$\:\pm\:$3.55
Mean value	$\:49.00\pm\:12.4$	97.54$\:\pm\:$0.04	35.98$\:\pm\:$0.60	28.30$\:\pm\:$2.76

**Fig. 3:**
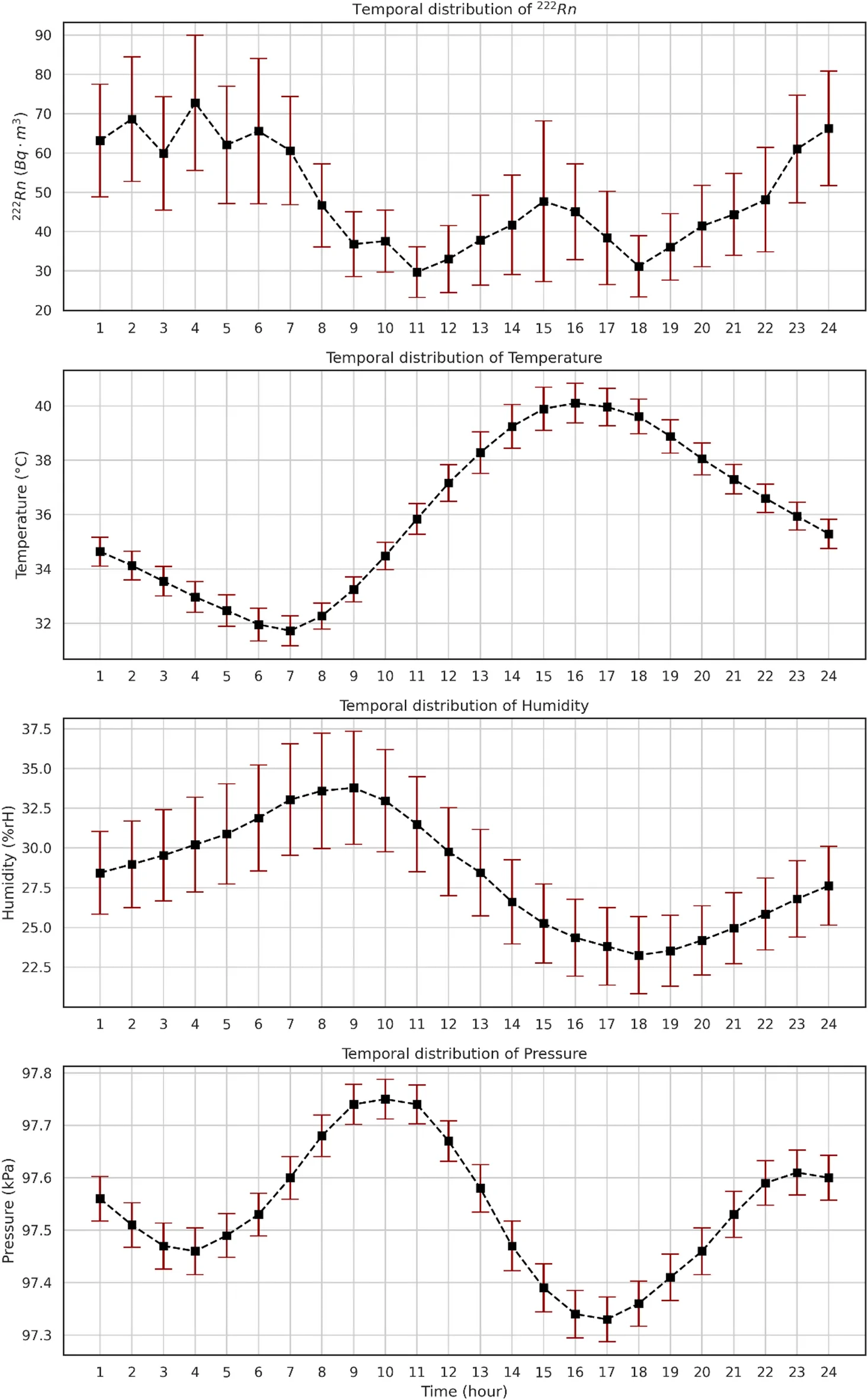
Temporal variation of average radon concentration, temperature, pressure and humidity values expressed with expanded uncertainty (k = 2 and 95% confidence interval) from mean values of 21 dwellings from Villy.

Figure 3 illustrates the temporal distribution of radon and atmospheric parameters. For radon concentration, three local maxima were recorded at 4:00 AM, 3:00 PM and midnight respectively, and two local minima were recorded at 11:00 AM and 6:00 PM respectively. Radon temporal distribution are subdivided into three parts. From midnight to 7:00 AM, concentration varied little and reached the daily maximum values. Between 7:00 AM and 6:00 PM, concentration decreased sharply and reached the daily minimum values before increasing sharply from 6:00 PM to midnight. This increase can be explained by the practice of keeping doors and windows closed in most dwellings at afternoon while the decrease is justified by opening doors and windows at morning.

The radon accumulation process occurs while occupants are inside the dwellings, while its release takes place when occupants are outside. This could be explained by the living habits. During the night doors and windows are closed, promoting radon accumulation and an increase in pressure and humidity due to occupants’ perspiration and respiration, as well as the lack of indoor air renewal. Doors and windows are opened around 5 a.m., allowing radon to escape, temperature to increase, pressure and humidity to decrease.

It is noted that when radon increases and stabilizes at maximum (between midnight and 7 a.m. and between 6 p.m. and midnight), the temperature decreases and the humidity increases. When the radon concentration decreases and reaches minimum, the temperature increases and the humidity decreases. This phenomenon is observed in the work of^27^, which shows that when the external temperature increases, the internal radon concentration decreases, with a moderate correlation coefficient of R² = 0.26 for linear regression. This study was conducted at much lower temperatures (around − 30 °C to 20 °C) than those of the present study.

## Statistical analysis

**Fig. 4:**
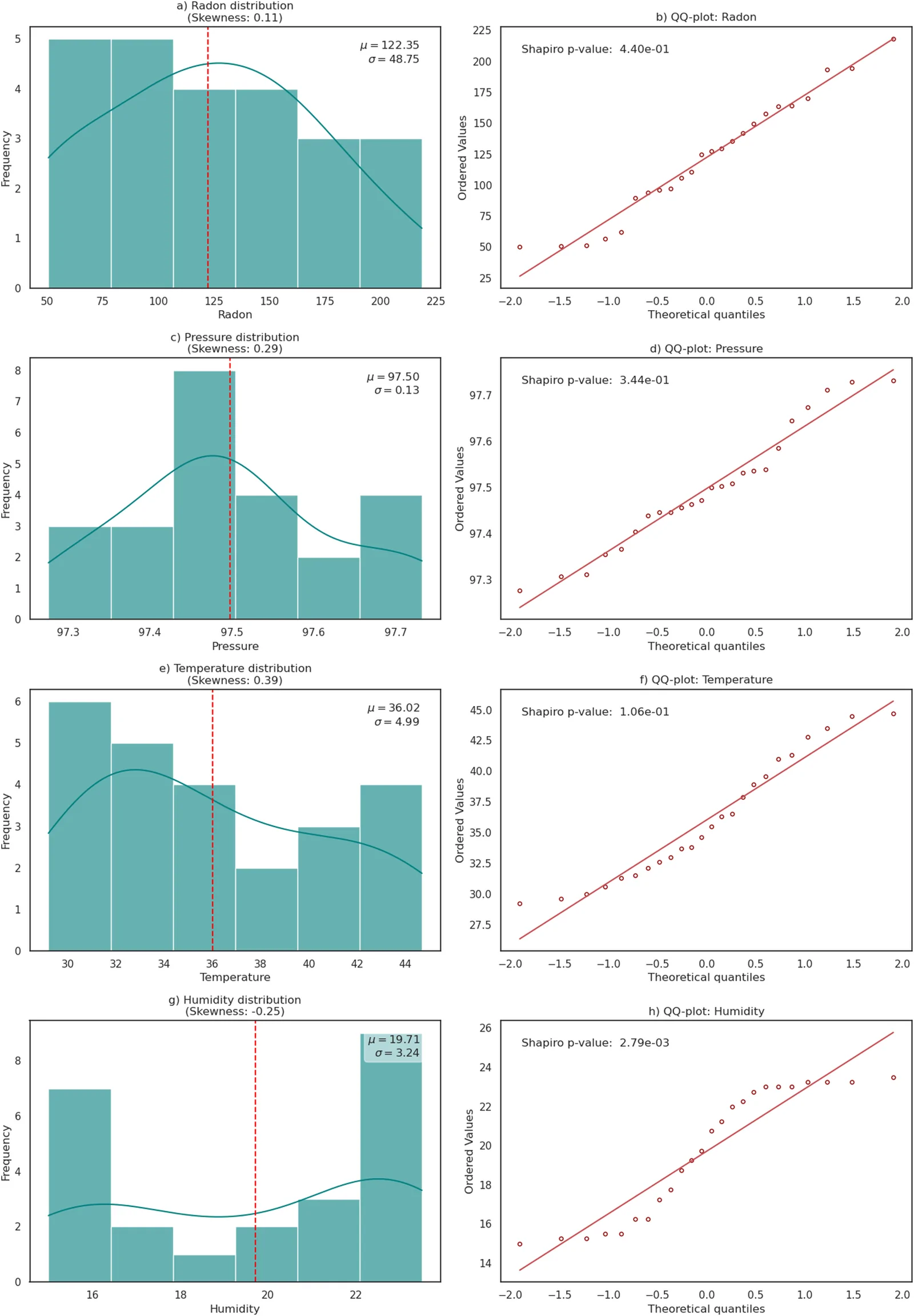
Shapiro-Wilk normality test of indoor radon concentration (**a**) and atmospheric parameters (**c**, **e**, **g**) with QQ-plot of indoor radon concentration in $\:Bq\bullet\:{m}^{-3}$ (**b**) and atmospheric parameters (**d**, **f**, **h**).

The Shapiro-Wilk test was performed on 24 hourly averaged data for two-day measurements radon concentration, temperature, pressure, and humidity of a single dwelling. Figures 4a, c, e, and g show the frequency distribution diagrams for radon, pressure, temperature, and humidity, respectively. The skewness calculation reveals an approximately normal distribution for all the variables. The skewness values are 0.11, 0.29, 0.39, and − 0.25 for radon, pressure, temperature, and humidity, respectively. This shows that all the variables have a near-normal distribution with a skewed distribution towards higher values except for the humidity, which has a skewed distribution towards lower values. This skewness is also observed in the work of^28^ for the radon concentration. Most of dwellings in the study area do not have mechanical ventilation systems. This can lead to the accumulation of radon gas inside and explain the positive skewed distribution observed in the radon data. The Shapiro-Wilk test has been performed on the same data and results show that the p-values are well above the reference value of 0.05 for radon, pressure and temperature, indicating that there is insufficient evidence to reject the null hypothesis (Fig. 4). Figures 4b, d and f of qq-plots show a normal acceptable approximation for the radon, pressure and temperature distributions, with a slight offset at the extremes. This is not the case for the humidity, whose qq-plot is shown in Fig. 4h.

**Fig. 5:**
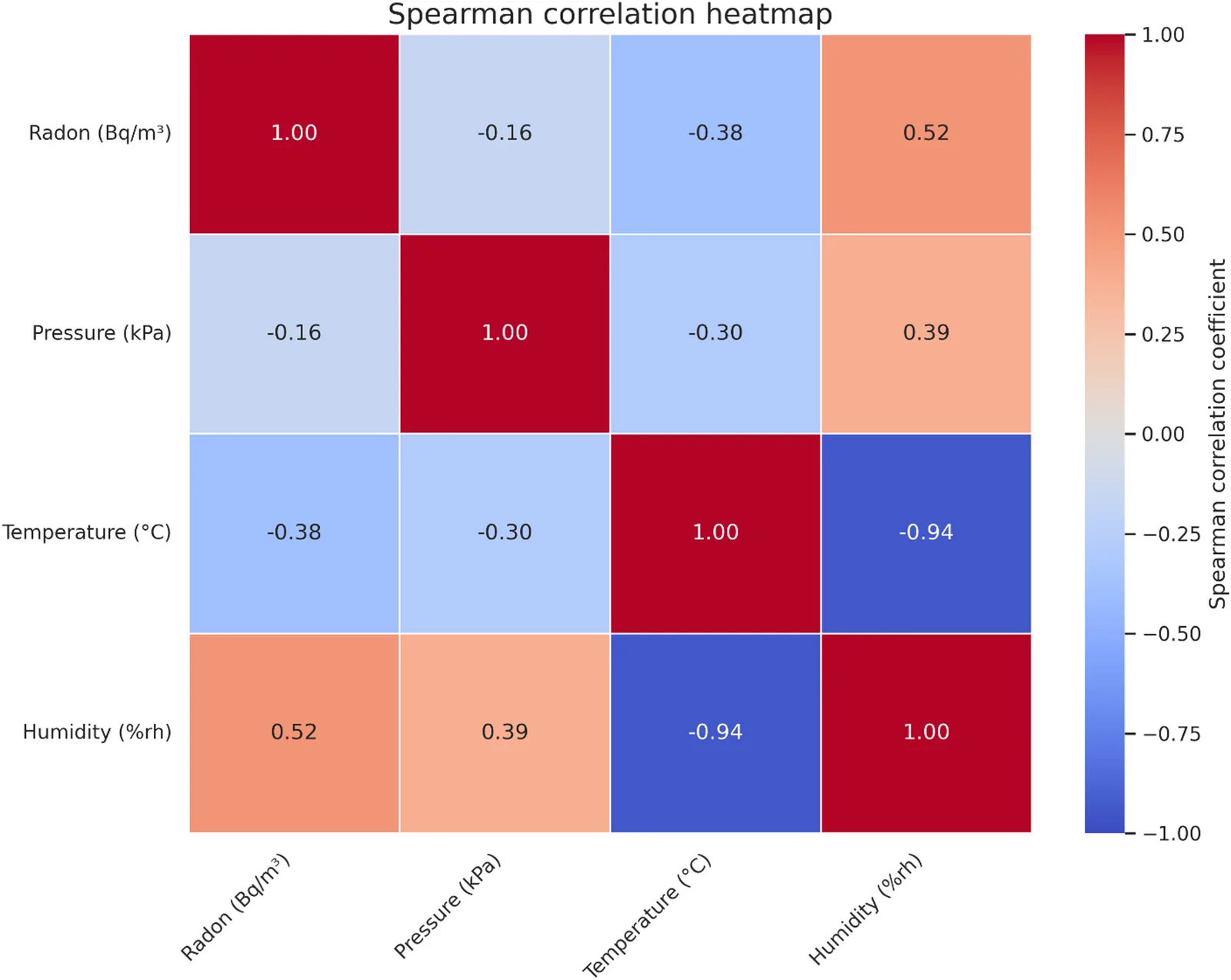
Spearman correlation heatmap showing relationship between radon and atmospheric parameters.

Figure 5 shows the Spearman correlation coefficients between all the variables. A very strong negative correlation is observed between humidity and temperature, with a Spearman correlation coefficient of *r* = -0.94 (Fig. 5). The inside temperature increase (decrease) when the inside humidity decrease (increase). Weak negative correlations are observed between radon and temperature, pressure and temperature, pressure and humidity, with Spearman correlation coefficients of *r* = -0.38, -0.30, and 0.39, respectively. This shows that when the temperature increases (or decreases), the radon concentration decreases (or increases). When pressure increases, the temperature decreases and the radon concentration increases. When pressure increases, the humidity increases. When radon, pressure, and humidity change in one direction (increase or decrease), the temperature changes in the opposite direction. These calculations confirm the observations made on previous section. A moderate positive correlation is observed between radon a humidity with a Spearman correlation coefficient of *r* = 0.52 which confirm that radon and humidity increase (or decrease) together.

### Study limitations

The present study has a significant limitation. Short-term radon measurements were taken inside each dwelling, covering only a 48-hour period. This does not allow for an effective representation of the actual radon concentration throughout the year, which is necessary for radiological risk assessment. Nevertheless, it provides an initial approach to studying radon, its diurnal evolution, and its influence from meteorological parameters. This study was conducted during a period that is expected to have minimal radon concentrations, yet relatively high concentrations were recorded. This highlights the need for more comprehensive research covering all seasons to obtain much more accurate data.

The most likely sources of measurement error in this study are temperature and dust. In fact, the maximum recorded temperature (38.8 °C) and the average temperature (35.98 °C) are both quite high. High temperatures can increase thermal noise in the electronic circuits and disrupt measurement accuracy. They can also influence radon movement through convection currents, even in the absence of a ventilation system. Radon can thus be rapidly moved towards or away from the monitor, leading to fluctuations in its concentration and resulting in unpredictable behaviour (like null values). In the environmental context of Burkina Faso, dust could settle inside the diffusion chambers, thus obstructing the accurate detection of alpha particles emitted by radon. In addition to these sources of error, there are certain difficulties related to radon measurement due to human activity.

It would have been preferable to study radon behaviour without the influence of human activity, such as opening and closing doors and windows. It also often happens that the monitor is moved by residents for some reason. This is identified and reported on the platform, and when it is noticed that this has affected the data, the corresponding dwelling is removed from the data. It should also be noted that despite having authorization from the national authorities to conduct this study, as well as permission from the village chief, some people were reluctant for taking measurements inside their homes.

## Conclusion

Analysis of the spatial distribution of radon concentration inside dwellings reveals significant variability, characterized by a standard deviation of $\:25.19\:Bq\cdot\:{m}^{-3}$. This highly heterogeneous nature makes the influence of atmospheric parameters unpredictable, as they fluctuate considerably. Radon concentration varies greatly from one dwelling to another, allowing them to be classified into three categories in the present study. The first category comprises 67% of dwellings with low radon concentrations. The second category consists of 28% dwellings with medium radon concentrations, and the final category, 5% dwellings, comprises high concentrations. To achieve the main objective of the present study, the temporal distribution of radon concentration in relation to atmospheric parameters was analysed. This analysis allowed to observe the evolution of radon concentration with atmospheric parameters. The highest radon concentrations during the day are measured between midnight and 7 a.m. and then between 6 p.m. and midnight. During this period, the temperature is also at minimum and the humidity at maximum. Conversely, the lowest radon concentrations are reached between 7 a.m. and 6 p.m., and during this period of day, the temperature rises to its maximum and the humidity falls to its minimum. So the increase in radon concentrations inside dwellings correspond to a decrease in temperature and an increase in humidity, and the decrease of radon concentrations correspond to an increase in temperature and a decrease in humidity. The study of correlations established a relationship between pressure and humidity. When pressure increases, humidity increases, and when pressure decreases, humidity decreases. Radon activity concentration does not pose a particular danger inside dwellings in the village of Villy, but it can be significant at times and in certain locations due to the heterogeneous nature of its spatiotemporal distribution. Residents of Villy are encouraged to well ventilate dwellings by keeping doors and windows open during certain periods of the day very often.

The present study is a preliminary one. It enabled short-term radon measurements to observe a trend in the influence of atmospheric parameters on its concentration. This entailed constraints resulting in a reduced measurement time. The average radon concentrations measured are significantly higher than those obtained in several other studies. This provides a valid reason to expand the present study by increasing the number of dwellings and the measurement time, potentially covering the entire year using a passive radon measurement method.

## Data Availability

The datasets generated during and/or analysed during the current study are available from corresponding author on reasonable request.
